# Surface Quality and Compressive Properties of Mortise and Tenon Lattice Structures Fabricated by Fused Deposition Modeling

**DOI:** 10.3390/ma18030628

**Published:** 2025-01-30

**Authors:** Bin Li, Byung-Won Min, Hai Gu, Jie Jiang, Jie Zhang, Hao Zhang

**Affiliations:** 1 School of Mechanical Engineering, Nantong Institute of Technology, Nantong 226002, China; libin19@ntit.edu.cn (B.L.); jiangjie@ntit.edu.cn (J.J.); zhangjie@ntit.edu.cn (J.Z.); zhanghao@ntit.edu.cn (H.Z.); 2 Jiangsu Key Laboratory of 3D Printing Equipment and Application Technology, Nantong Institute of Technology, Nantong 226002, China; 3 Division of Information and Communication Convergence Engineering, Mokwon University, Daejeon 35349, Republic of Korea; minfam@mokwon.ac.kr

**Keywords:** fused deposition modeling, mortise and tenon, lattice structure, surface quality, compressive properties

## Abstract

To address the anisotropy of mechanical properties and the challenge of removing support materials in lattice structures fabricated using fused deposition modeling (FDM), this study is inspired by traditional woodworking mortise and tenon joints. A hexagonal interlocking mortise lattice structure was designed, and mortise and tenon lattice structures (MTLSs) with various parameters were fabricated. Compared with the traditional integrated forming lattice structure (IFLS), the MTLS exhibits maximum reductions in side surface roughness (Ra), printing time, and material consumption of 74.87%, 25.55%, and 52.21%, respectively. In addition to enhancing surface quality and printing efficiency, the MTLS also exhibited superior mechanical properties. The uniaxial compression test results show that the specific strength, energy absorption (EA), and specific energy absorption (SEA) of the MTLS exhibit maximum increases of 51.22%, 894.59%, and 888.39%, respectively, compared with the IFLS. Moreover, the effects of strut angle and thickness on the lattice structure were analyzed. Smaller strut angles and larger strut thicknesses endowed greater strength, while smaller angles contributed to higher energy absorption. This study proposes a novel approach for designing lattice structures in additive manufacturing.

## 1. Introduction

Lattice structures, characterized by their high specific surface area, lightweight nature, and high specific strength and stiffness [1], have demonstrated great potential in energy absorption, thermal insulation, load bearing, noise reduction, and vibration damping [2,3,4,5], thus garnering significant interest. Commonly used materials include metals (such as stainless steel [6], titanium alloys [7], aluminum alloys [8], nickel-based alloys [9]), polymers (such as polylactic-acid (PLA) [10], acrylonitrile–butadiene–styrene (ABS) [11], polyether ether ketone (PEEK) [12], thermoplastic polyurethane (TPU) [13]), ceramics (such as Al_2_O_3_ [14], SiC [15]), and composites (such as carbon fiber (CF) composites [16] and glass fiber (GF) composites [17]). These materials can provide excellent performance combinations based on different application requirements. Currently, lattice structures have been applied widely in fields such as aerospace, biomedical engineering, the automotive sector, and sports equipment. In the aerospace sector, lattice structures are used to manufacture lightweight structural components for aircraft and rockets [18]; in the biomedical field, they are utilized to produce implants that bond more rapidly and securely with bone tissue [19,20]; in the automotive industry, they are employed to create lightweight components [21,22]; and in sports apparatus, lattice structures are applied to produce protective gear [23,24].

Traditional methods for fabricating lattice structures primarily include investment casting [25], extrusion cutting [26], stamping [27], stretched mesh folding [28], and joint assembly [29]. Investment casting, extrusion cutting, stamping, and stretched mesh folding necessitate the use of specialized molds and intricate preparation procedures, thereby escalating costs. Additionally, the investment casting method places stringent demands on the molten metal’s fluidity and is prone to defects. Extrusion cutting and stamping processes entail repeated material removal, resulting in substantial waste. The joint assembly method depends on conventional machining technologies, with geometric shapes limited by the capabilities of manufacturing equipment and processes [30].

Additive manufacturing (AM), also known as 3D printing, offers exceptional design flexibility, high material efficiency, low manufacturing costs, and the ability to fabricate complex structures [31]. These advantages effectively address the challenges associated with the fabrication of intricate lattice structures, opening new avenues for their development and application [32]. Thales Alenia Space (Rome, Italy), in collaboration with the European Space Agency (ESA, Paris, France), implemented additive manufacturing techniques involving lattice structures for deployable solar panel systems, reducing manufacturing costs by 75% [33]. The Qiansheng-1 satellite (China Academy of Space Technology, Beijing, China), launched in 2019, marked the inaugural application of additive manufacturing to fabricate three-dimensional lattice structures, shortening the design and manufacturing cycle to one month [34].

Different AM methods intrinsically differ significantly, which plays a crucial role in determining the surface characteristics and mechanical properties of the fabricated structures. In powder bed fusion techniques such as selective laser sintering (SLS) and selective laser melting (SLM), residual unsintered powder particles often remain on the surface, resulting in a rough and porous microstructure. The uneven distribution and size of the powder particles contribute to variations in surface roughness, chemical reactivity, and wettability, and influence the mechanical properties of the final component [35]. In resin-based techniques like stereolithography (SLA) and digital light processing (DLP), the curing process of the resin can result in slight surface irregularities, leading to distinct surface features and unique mechanical behavior due to the degree of curing [36]. In fused deposition modeling (FDM), where filament materials are extruded and deposited layer by layer, the surface roughness shows directional dependence, which in turn affects the mechanical performance of the printed structure [13].

FDM, a widely used AM technology, is known for its low cost and simple operation [37,38,39]. Lattice structures are generally produced as integrated printing fabricated via FDM [40,41,42]. Calise et al. [43] explored the mechanical behavior and anisotropy of octahedral lattice structures with two different relative densities made from ABS and demonstrated that the stiffness and strength of the printed components exhibit anisotropy. Gautam et al. [44] analyzed the compressive performance of Kagome truss lattices made from ABS and revealed that altering the building direction increased the average peak strength and effective stiffness by 23% and 19%, respectively. Lazar et al. [45] fabricated triply periodic minimal surface (TPMS) lattice structures with varying relative densities using short glass fiber-reinforced polyamide and verified that their compressive performance was significantly affected by anisotropy. Ursini et al. [13] explored the mechanical behavior of TPU lattice structures and found that the layer stacking process caused anisotropy in stiffness, which made it difficult to predict. Wang et al. [46] proposed a design optimization method for heterogeneous conformal lattice structures based on principal stress lines (PSL) and developed an optimization model that takes into account anisotropy and constraints.

Changing the building direction can effectively enhance the mechanical performance of lattice structures [47,48,49]. However, this approach often results in the generation of substantial support material. The presence of support materials not only increases printing time and the cost of material consumption but also complicates its removal, especially for complex lattice structures [50]. Liu et al. [51] addressed this challenge by introducing the interlocking assembly method into FDM lattice fabrication. This method enabled the production of typical body-centered cubic (BCC) lattice structure, enhanced their mechanical properties, and removed the necessity for support printing.

The mortise and tenon structure is a primary connection method used in ancient Chinese architecture, furniture, and other wooden instruments. Renowned for its structural stability, excellent mechanical properties, capacity to withstand high loads, good seismic resistance, and disassemblability, this structure exemplifies a long history and unique craftsmanship value [52]. The mortise and tenon structure connects two wooden components using complementary convex and concave sections. The protruding part is called the tenon (or tenon head), and the recessed part is referred to as the mortise (or mortise hole or mortise slot). This structure effectively restricts the twisting of wooden components in all directions, ensuring a secure connection. Various types of mortise and tenon structures exist, including straight tenons, dovetail tenons, cross tenons, running horse tenons, shoulder tenons, and hexagonal intersecting tenons. Li et al. [53] used FDM to fabricate 2D honeycomb structures with dovetail connections and different module infill patterns, analyzing the influence of geometric parameters on failure modes and energy absorption characteristics. Xu et al. [54] applied dovetail joints to honeycomb panels, designing and fabricating different 2D dovetail-connected honeycomb structures using FDM, and studied the effects of connection methods on energy absorption and deformation behavior. Yan et al. [55] utilized DLP to produce 2D honeycomb structures based on straight tenons and investigated the sound absorption properties of the modified honeycomb structures. Currently, research on mortise and tenon joints in lattice structures remains limited to 2D honeycomb structures using dovetail or straight tenon connections, with no studies reported on 3D mortise and tenon lattice structures (MTLSs).

This study integrates traditional mortise-and-tenon joints with FDM technology, resulting in a novel three-dimensional lattice structure. The structure is constructed by assembling pre-decomposed two-dimensional components produced using FDM. The effects of different joint angles and strut thicknesses on the surface quality and mechanical properties of the lattice structure are analyzed, with comparisons drawn against lattice structures fabricated via FDM integrated forming lattice structures (IFLS). Furthermore, the relationships between the relative density of MTLS and printing time, material consumption, compressive performance, and energy absorption characteristics are examined, resulting in predictive models. This research enhances surface quality, reduces printing time and material consumption, and significantly improves structural strength and energy absorption characteristics. It offers theoretical and practical insights to guide the application of additively manufactured lattice structures.

## 2. Materials and Methods

### 2.1. Lattice Structure Design

**Figure 1:**
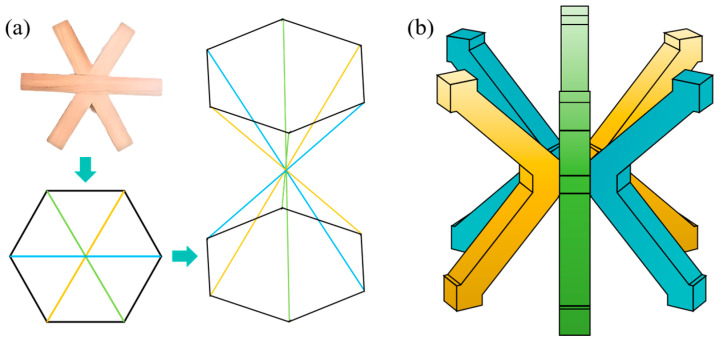
MTLS: (**a**) design approach for MTLS; (**b**) 3D model of the MTLS.

The hexagonal intersecting tenon is a complex form of mortise and tenon joint designed to securely connect six wooden components at a single junction. It is widely applied in traditional architecture, furniture, and wooden tools, especially suitable for three-dimensional structures with multiple-angle intersections. This study employs the typical hexagonal intersecting tenon, characterized by six edges in appearance, formed by three square components inclined at a 60° angle to each other. Figure 1a shows the lattice structure inspired by the hexagonal intersecting tenon. The core of the designed MTLS incorporates a hexagonal intersecting tenon mortise structure. During assembly, each square component’s joining section retains one-third of its original thickness, as shown in Figure 1b, the struts correspond to those in Figure 1a through color.

**Figure 2:**
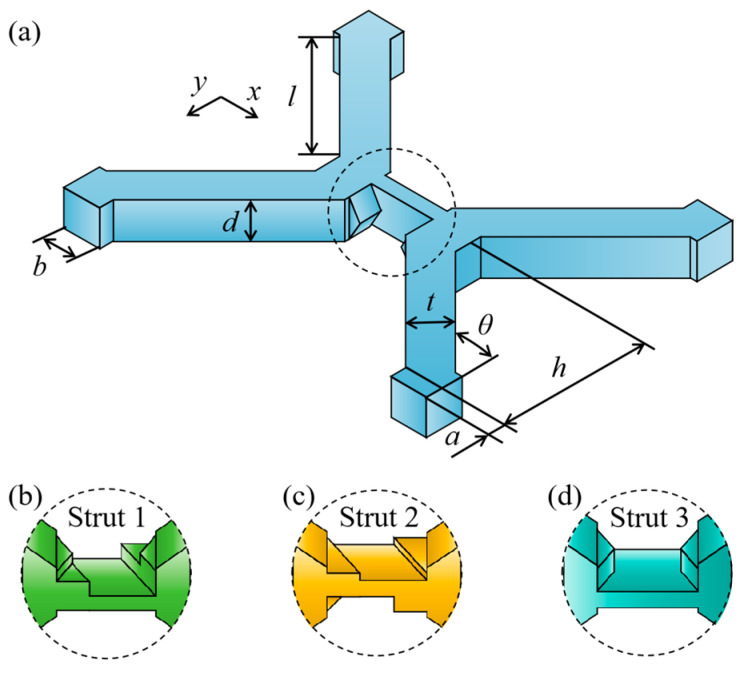
Schematic diagram of the MTLS: (**a**) MTLS parameters; (**b**) Strut 1 structure; (**c**) Strut 2 structure; (**d**) Strut 3 structure.

The designed MTLS comprises three struts with a similar basic structure, as shown in Figure 2a. Key parameters include the strut thickness (*t*), the edge length of the top plane (*b*), the pillar width (*d*), the height from the strut center to the plate (*h*), and the angle between the pillar and the plane (*θ*). Notably, *t* = *b* = *d*, and *h* = 20 mm. The primary distinction among the three struts lies in the adoption of the hexagonal intersecting tenon mortise structure, which introduces variation and results in inconsistency, as depicted in Figure 2b. The parameters of the designed MTLS are detailed in Table 1.

The relative density (RD) of the lattice structure is defined as the ratio of the actual volume (*V*_1_) of the model structure with solid unit filling to the solid volume (*V*) of the original model:

RD = *V*_1_/*V* (1)

**Figure 3:**
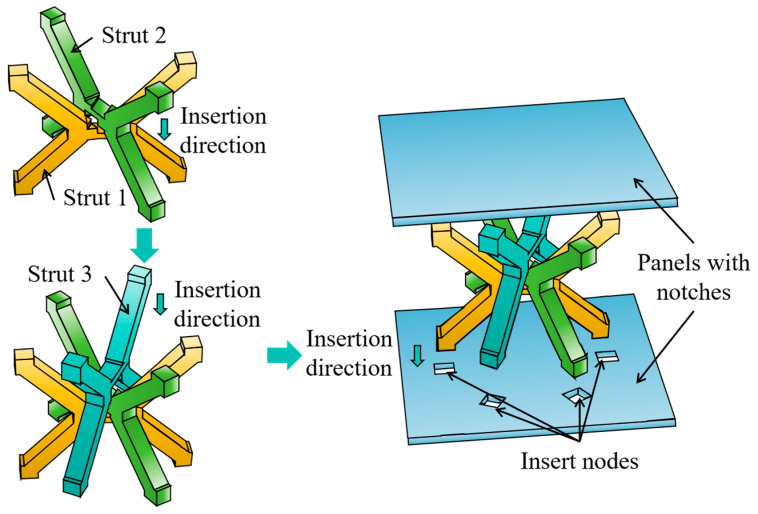
Schematic diagram of the MTLS assembly.

The designed MTLS is assembled following the installation steps of the hexagonal intersecting tenon. Additionally, the sandwich panels are mounted on the top and bottom of the MTLS, as illustrated in Figure 3.

### 2.2. Lattice Structure Analytical Model

During the compression process, the MTLS may exhibit three possible failure modes.

At high relative densities, the lattice structure struts yield and fail, and the peak strength of the lattice structure [16,56] is

(2) $$\sigma_{pk}=6\cos\theta \frac{t^{2}}{A}\sigma_{y}$$

where *σ_y_* is the compressive yield strength of the material. *A* is the cross-sectional area of the MTLS, as shown in Figure 2a.

(3) $$A={\left[40\tan\theta +(2+\sqrt{3})t\right]}^{2}$$

At lower relative densities, the struts are prone to elastic buckling failure. Substituting the yield strength of the strut material with the elastic buckling stress yields the lattice structure strength *σ_E_*:

(4) $$\sigma_{E}=\frac{k^{2}\pi^{2}E_{s}}{4800}{\left(t\cos\theta \right)}^{2}$$

where *E*_s_ is the elastic modulus of the material, and *k* is determined by the end conditions of the buckling support plate: *k* = 1 or 2 for pin-jointed or built-in end conditions, respectively. If one end of the strut is pin-jointed and the other is a built-in condition, *k*^2^ ≈ 2.

At moderate relative densities, the compressive strength of the lattice is governed by inelastic buckling. According to the Shanley–Engesser tangent modulus theory [56], the elastic modulus *E_s_* is replaced by *E_t_*, yielding the inelastic buckling stress *σ_IE_*.

(5) $$\sigma_{IE}=\frac{k^{2}\pi^{2}E_{t}}{4800}{\left(t\cos\theta \right)}^{2}$$

where *E_t_* is the tangent modulus of the stress–strain curve.

### 2.3. Lattice Structure Fabrication

The lattice structure was fabricated using an FDM printer (UP 300, Taier Times, Beijing, China). ABS+ filament (eSUN, Guanghua Weiye, Shenzhen, China) was selected as the printing material, with its performance parameters detailed in Table 2. ABS+ is a modified version of traditional ABS, offering enhanced mechanical properties and reduced shrinkage. This material exhibits high toughness and impact resistance, making it ideal for printing durable components that are less susceptible to warping or cracking during the printing process.

**Figure 4:**
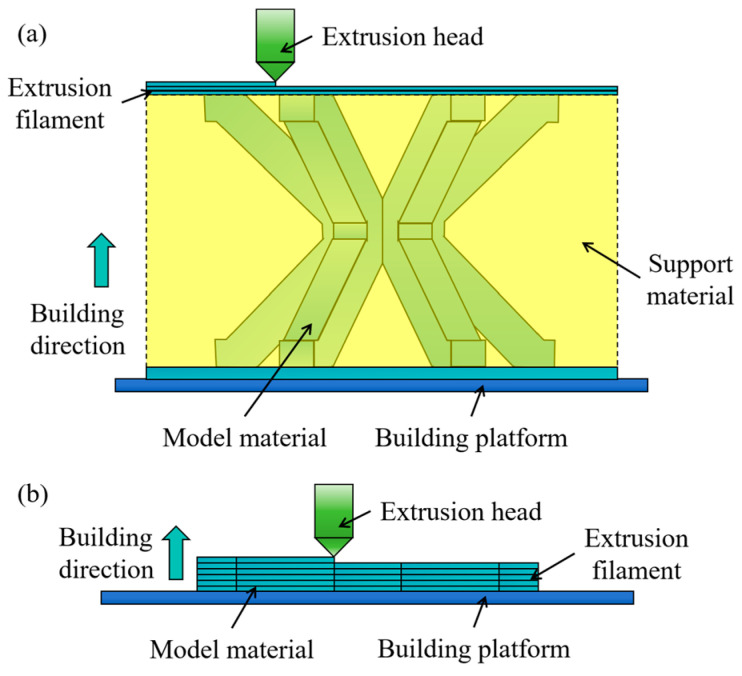
Schematic diagram of the two forming methods: (**a**) IFLS; (**b**) MTLS.

To compare the surface quality and compressive performance of the designed MTLS, an IFLS identical to the assembled MTLS was designed using the general method of FDM-fabricated lattice structures. The printing orientation is illustrated in Figure 4a. During fabrication, the MTLS and sandwich panel were printed separately, as shown in Figure 4b. Based on the printer’s resolution, the mortise and slot sections have a reserved tolerance of 0.1 mm to ensure proper matching of the three struts and the sandwich panels. After printing, the components were assembled according to the sequence shown in Figure 3, and adhesive was applied at the joints to reinforce the structural integrity of the MTLS.

**Figure 5:**
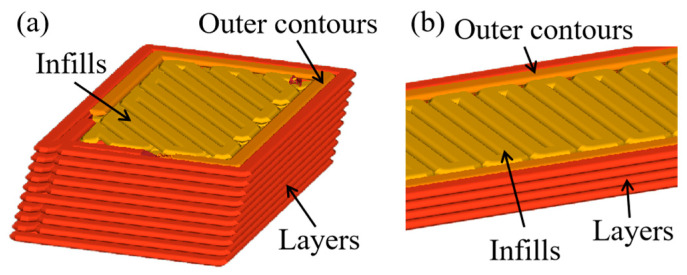
Schematic diagram of print profiles: (**a**) IFLS; (**b**) MTLS.

The parameter design and slicing were performed using the printer’s built-in software, UP Studio3. Detailed printing specifications are provided in Table 3, with the infill method and other settings configured according to the printer’s default parameters. The print profile is depicted in Figure 5.

### 2.4. Lattice Structure Performance Tests

**Figure 6:**
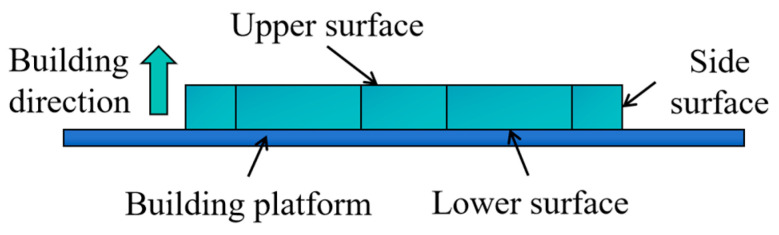
Measurement locations for surface morphology and surface roughness.

To accurately evaluate the forming quality and performance of the lattice structure, measurements and characterization are essential. The surface morphology of the fabricated struts was examined using an image-measuring instrument (VMS322, QTTEK, Changzhou, China). The surface in contact with the printing platform is referred to as the bottom, while the opposite surface is considered the top. The building direction is designated as the side surface. The measurement schematic is shown in Figure 6. The integrated forming area corresponds to the region after the individual components are assembled. Surface roughness (Ra) for each surface was measured using a surface roughness tester (SJ-210, Mitutoyo, Kawasaki, Japan). Each surface was measured five times, and the average value was taken as the final result.

The printing time displayed on the printer is considered the model’s printing time, with the MTLS time being the sum of the printing times for the struts and core panels. The mass of the MTLS struts, core panels, and IFLS components, both before and after support removal, was measured using a precision electronic balance (LAB 214e, ADAM EQUIPMENT, Wuhan, China) with an accuracy of 0.0001 g.

**Figure 7:**
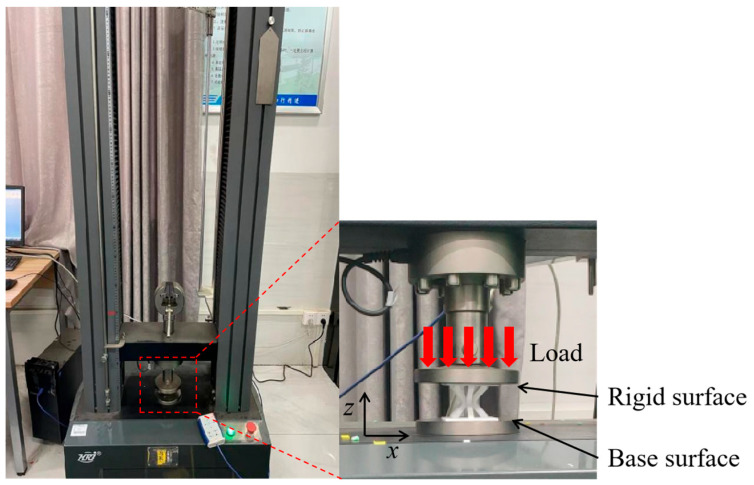
Schematic diagram of compression test.

To evaluate the effectiveness of the lattice structure design strategy, it is essential to obtain the corresponding mechanical response data for the lattice structure. A universal testing machine (WDW-50, Hengruijin, Jinan, China) is employed to perform a compression test on the fabricated lattice structure. At room temperature, a load is applied along the Z-axis at a constant rate of 1 mm/min. During the compression test, the loading process was terminated when the force dropped to 20% of the peak value, as shown in Figure 7. An industrial camera was used during the experiment to record the deformation process of the sample.

## 3. Results and Discussions

### 3.1. Surface Morphology and Surface Roughness

**Figure 8:**
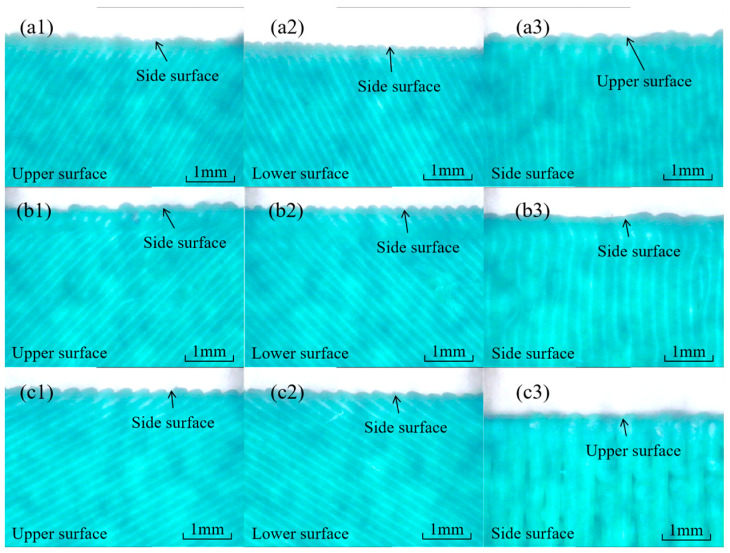
Surface morphology of the IFLS: (**a1**) upper surface, *θ* = 30°; (**a2**) lower surface, *θ* = 30°; (**a3**) side surface, *θ* = 30°; (**b1**) upper surface, *θ* = 45°; (**b2**) lower surface, *θ* = 45°; (**b3**) side surface, *θ* = 45°; (**c1**) upper surface, *θ* = 60°; (**c2**) lower surface, *θ* = 60°; (**c3**) side surface, *θ* = 60°.

**Figure 9:**
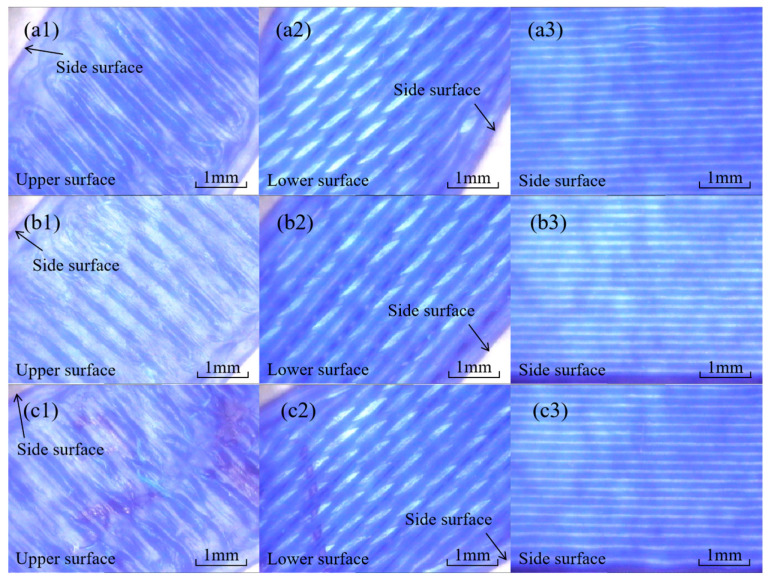
Surface morphology of the MTLS: (**a1**) upper surface, *θ* = 30°; (**a2**) lower surface, *θ* = 30°; (**a3**) side surface, *θ* = 30°; (**b1**) upper surface, *θ* = 45°; (**b2**) lower surface, *θ* = 45°; (**b3**) side surface, *θ* = 45°; (**c1**) upper surface, *θ* = 60°; (**c2**) lower surface, *θ* = 60°; (**c3**) side surface, *θ* = 60°.

The surface morphology of the three surfaces of the IFLS and MTLS was observed using an image measuring instrument, with the results shown in Figure 8 and Figure 9, respectively. In these figures, the white areas represent the gaps between adjacent extruded filaments, while the dark areas indicate the extruded filament of the lattice structure.

As shown in Figure 8, distinct gaps exist between the deposition layers on each surface of the IFLS. The side surface exhibits a distinct stepped appearance, with the shape of the “staircase” varying according to the value of *θ*. The surface of the IFLS is relatively smooth, as there is no angle of inclination between the deposition layers and the measured surface.

As shown in Figure 9, noticeable gaps are present between the fill filaments on the surface and bottom of the MTLS, which is consistent with the printing path shown in Figure 5b. The side surface of the MTLS is relatively smooth; however, the gaps between the layers are clearly visible.

**Figure 10:**
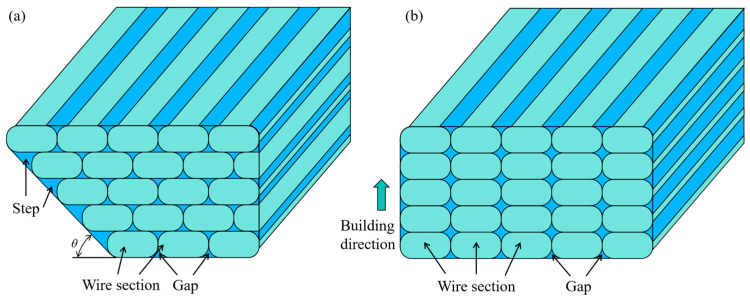
Schematic of the lattice structure forming cross-section: (**a**) IFLS; (**b**) MTLS.

Figure 10 illustrates the cross-sectional schematic of lattice structures formed by different methods. As depicted in the figure, the filament cross-sections do not align perfectly, which leads to gaps. In the IFLS, the deposition layers are distributed sequentially along the angle of inclination of the support, creating a staircase surface, as shown in Figure 10a. In contrast, the filament in the MTLS is deposited along the building direction, with noticeable gaps visible on the surface of the struts, as shown in Figure 10b.

**Figure 11:**
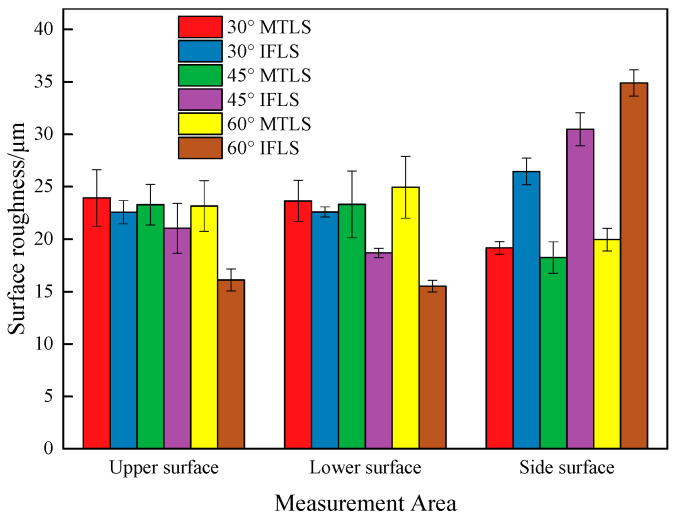
Surface roughness values of different regions of the *t* = 6 mm lattice.

The measured surface roughness (Ra), values are presented in Figure 11. As shown in the figure, the surface and bottom Ra values of the MTLS are nearly identical. The average Ra of the surface is 23.46 μm, which is larger due to the non-integer ratio between the strut thickness and filament width, leading to the formation of gaps. The average Ra of the bottom is 23.97 μm, primarily because a base is required during the forming process, and the bottom surface is in contact with the base. After the base is removed, the Ra of the bottom surface increases. The average Ra of the side is 19.13 μm, which is lower due to the smaller gaps between the layers. For the IFLS, the surface and bottom Ra values are nearly identical, with average values of 19.91 μm and 18.93 μm, respectively.

The Ra values of the side surfaces of both the MTLS and the IFLS increase with *θ*, which is consistent with the conclusion of the “staircase” effect. The side Ra values of the IFLS are higher by 37.98%, 66.98%, and 74.87% compared with the MTLS at different *θ* values.

### 3.2. Printing Time and Material Consumption

**Figure 12:**
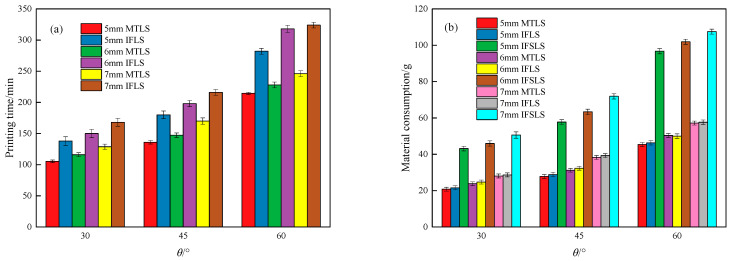
Printing time and material consumption of lattice structures: (**a**) printing time; (**b**) material consumption.

The cost of forming lattice structures using FDM technology is primarily reflected in printing time and material consumption. Figure 12 compares the printing time and material consumption of MTLS and IFLS at different *θ* and *t* values.

From Figure 12a, it can be observed that as both *t* and *θ* increase, the printing time gradually rises. This is due to the increased relative density of the lattice structure, as shown in Table 1. Compared with the IFLS, the printing time of the MTLS with different *θ* values was reduced by 23.25%, 23.78%, and 25.44%, respectively, while the printing time of MTLS with different *t* values was reduced by 22.90%, 24.03%, and 25.55%, respectively. As seen in Figure 4, the IFLS requires a significant amount of support structures during the forming process to ensure smooth printing, which greatly increases the printing time. In addition, the MTLS has a smaller size in the Z direction compared with the IFLS, while the cross-sectional area in the X–Y plane is larger. This reduces the number of times the print head moves in the forming direction, thereby decreasing the printing time.

As shown in Figure 12b, the material consumption of MTLS with different *θ* values was reduced by 48.17%, 49.75%, and 50.16%, respectively, compared with the IFLS. The material consumption of MTLS with different *t* values was reduced by 46.03%, 49.84%, and 52.21%, respectively. This is primarily because the IFLS requires a significant amount of support structures to ensure smooth printing, which leads to increased material consumption.

The IFLS has a higher model mass after the removal of support structures compared with the MTLS. This indicates that the “staircase” effect increases the mass of certain parts of the lattice structure, which is consistent with the conclusions drawn from the surface morphology observations.

**Figure 13:**
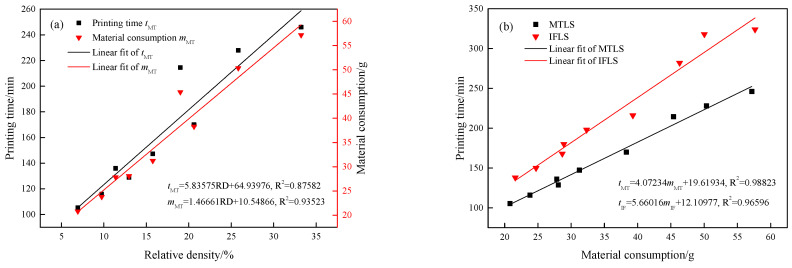
The relationship between printing time and material consumption of lattice structures: (**a**) the correlation among printing time, material consumption, and relative density of the MTLS; (**b**) the comparison of printing time and material consumption between the MTLS and IFLS.

Figure 13a compares the relationship between printing time, material consumption, and relative density of the MTLS. Figure 13b compares the printing time and material consumption of the MTLS with those of the IFLS. In this comparison, the material consumption of the IFLS refers to the lattice mass after the removal of support structures, while the printing time refers to the total processing time.

The relationship between the printing time (*t*_MT_), material consumption (*m*_MT_) and relative density (RD) of the MTLS is as follows:

*t*_MT_ = 5.83575RD + 64.93976 (6)

*m*_MT_ = 1.46661RD + 10.54866 (7)

It can be observed that the printing time, material consumption, and relative density of the MTLS all exhibit a linear relationship, with coefficients of determination (R^2^) of 0.87582 and 0.93523, respectively, indicating a good fit for the predictions.

The relationship between the printing time (*t*_MT_) of the MTLS, the printing time (*t*_IF_) of the IFLS, and their respective material consumptions (*m*_MT_ and *m*_IF_):

*t*_MT_ = 4.07234 *m*_MT_ + 19.61934 (8)

*t*_IF_ = 5.66016 *m*_IF_ + 12.10977 (9)

The printing time of lattice structures increases linearly with material consumption, with R^2^ of 0.98823 and 0.96596, suggesting a high degree of predictive accuracy. The slope of the predicted curve for the IFLS is steeper than that of the MTLS, indicating that, for the same print quality, the IFLS requires more time than the MTLS.

### 3.3. Compression Performance

**Figure 14:**
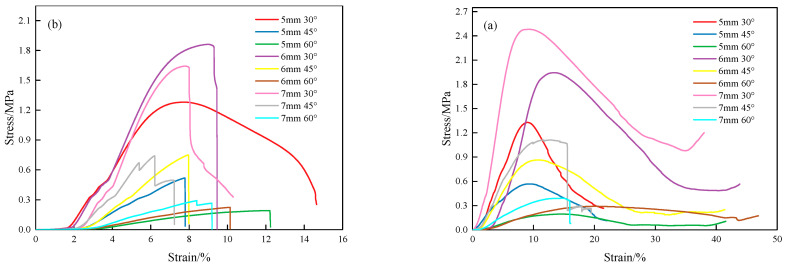
Stress–strain curve of the lattice structure: (**a**) IFLS; (**b**) MTLS.

The stress–strain curves of the lattice structure are obtained through the compression test, as shown in Figure 14. From Figure 14, it can be observed that the forming method significantly affects the fracture mode of the lattice. The first two stages of compression for both the MTLS and IFLS are essentially the same. In the first stage, the stress–strain curve increases linearly, with stress rising rapidly as *θ* decreases and *t* increases. In the second stage, elastic deformation occurs, and stress gradually decreases. In the third stage, the IFLS undergoes elastic deformation followed by immediate fracture without a distinct yielding phase, indicating brittle fracture, while the MTLS exhibits multiple fractures, accompanied by torsional deformation.

The specific strength *σ_b_* is the ratio of the material’s strength to its relative density. The higher the specific strength, the less material is required to achieve the corresponding strength.

*σ_b_* = *σ*_0_/RD (10)

where *σ*_0_ is the compressive stress.

The specific modulus *γ* is the ratio of the material’s elastic modulus to relative density. A higher specific modulus indicates a greater elastic modulus per unit volume, meaning the material will deform less under load, exhibiting greater stiffness.

*γ* = *E*/RD (11)

where *E* is the elastic modulus.

**Figure 15:**
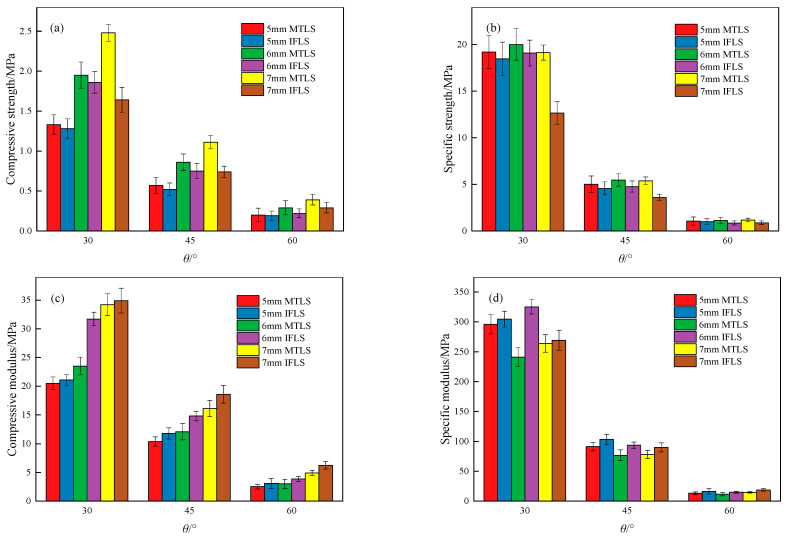
Comparison of lattice structure strength and modulus: (**a**) compressive strength; (**b**) specific strength; (**c**) compressive modulus; (**d**) specific modulus.

Combining Equations (6) and (7), the specific strength and specific modulus of the lattice structure can be obtained, as shown in Figure 15. From Figure 15, it is observed that as *θ* increases, the compressive strength, specific strength, compressive modulus, and specific modulus of the lattice structure gradually decrease. For the MTLS, compared with the 30° lattice, the compressive strength decreased by 84.27%, 84.96%, and 85.13% at *θ* = 60°, respectively. The specific strength decreased by 93.87%, 94.39%, and 94.52%, respectively. The compressive modulus decreased by 85.61%, 87.19%, and 87.71%, respectively. The specific modulus decreased by 94.39%, 95.17%, and 95.52%, respectively.

As *t* increases, the compressive strength and compressive modulus of the MTLS gradually increase, while the specific strength and specific modulus remain nearly unchanged. At *t* = 7 mm, the compressive strength increased by 86.47%, 94.74%, and 95.00%, and the compressive modulus increased by 54.81%, 66.83%, and 95.24%, compared with the values at *t* = 5 mm. The average specific strength at different *θ* values was 19.44 MPa, 5.28 MPa, and 1.12 MPa, respectively. The average specific modulus at different *θ* values was 226.94 MPa, 82.07 MPa, and 13.24 MPa, respectively.

Compared with the IFLS, the compressive strength and specific strength of the MTLS increased by 3.91% to 51.22%, while the compressive modulus and specific modulus decreased by 2.05% to 34.89%. For small values of *θ*, the lattice structure uses less material to achieve the corresponding strength and exhibits higher stiffness. The influence of *t* on the material used to achieve the corresponding strength and the stiffness of the lattice structure is generally minimal. When reaching the corresponding strength, the MTLS uses less material than the IFLS. The stiffness of the IFLS is greater than that of the MTLS.

**Figure 16:**
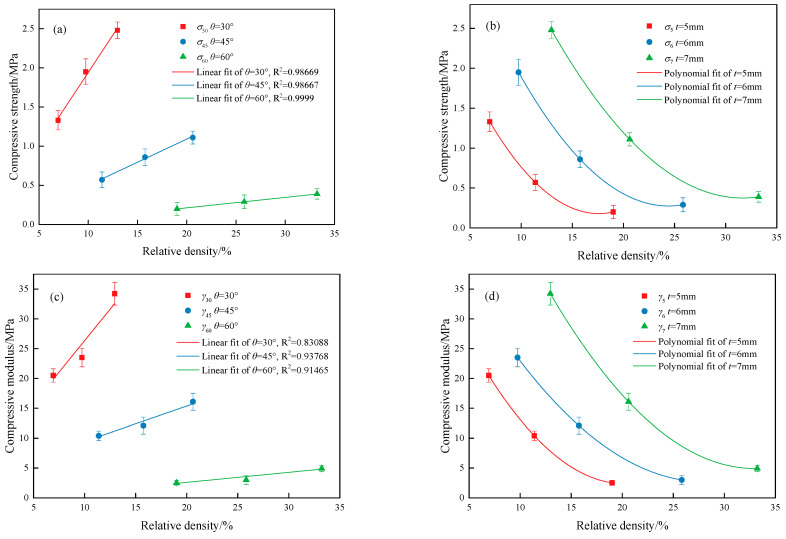
The compressive strength and compressive modulus of the MTLS and their relationship with relative density: (**a**) compressive strength at different *t*; (**b**) compressive strength at different *θ*; (**c**) compressive modulus at different *t*; (**d**) compressive modulus at different *θ*.

Figure 16 compares the relationship between the compressive strength, compressive modulus of the MTLS, and relative density. The relationship between compressive strength, compressive modulus, and relative density for MTLS with different *t* values, as obtained from Figure 16a,c, was fitted linearly. The R^2^ for the *σ*_30_, *σ*_45_, and *σ*_60_ fitting curves were 0.98669, 0.98667, and 0.9999, respectively, while for the *γ*_30_, *γ*_45_, and *γ*_60_ fitting curves, the R^2^ values were 0.83088, 0.93768, and 0.91465, respectively.

(12) $$\left\{\begin{array}{c} \sigma_{30}=0.1906RD+0.03629\\ \sigma_{45}=0.05916RD-0.09222\\ \sigma_{60}=0.01332RD-0.05334 \end{array}\right.$$

(13) $$\left\{\begin{array}{c} \gamma_{30}=2.1071RD+5.26514\\ \gamma_{45}=0.59227RD+3.5234\\ \gamma_{60}=0.16826RD-0.77123 \end{array}\right.$$

The relationship between compressive strength, compressive modulus, and relative density for MTLS at different *θ* values, as obtained from Figure 16b,d, was fitted using a polynomial.

(14) $$\left\{\begin{array}{c} \sigma_{5}=0.01009{RD}^{2}-0.35519RD+3.30707\\ \sigma_{6}=0.00776{RD}^{2}-0.37929RD+4.91051\\ \sigma_{7}=0.00601{RD}^{2}-0.38055RD+6.40308 \end{array}\right.$$

(15) $$\left\{\begin{array}{c} \gamma_{5}=0.10186{RD}^{2}-4.13063RD+44.23348\\ \gamma_{6}=0.06183{RD}^{2}-3.47401RD+51.49431\\ \gamma_{7}=0.07283{RD}^{2}-4.80866RD+84.28706 \end{array}\right.$$

In future research, the equations can be used to derive the compressive strength and compressive modulus of MTLS at different relative densities. Therefore, these equations can support the design of new structures in the future.

**Figure 17:**
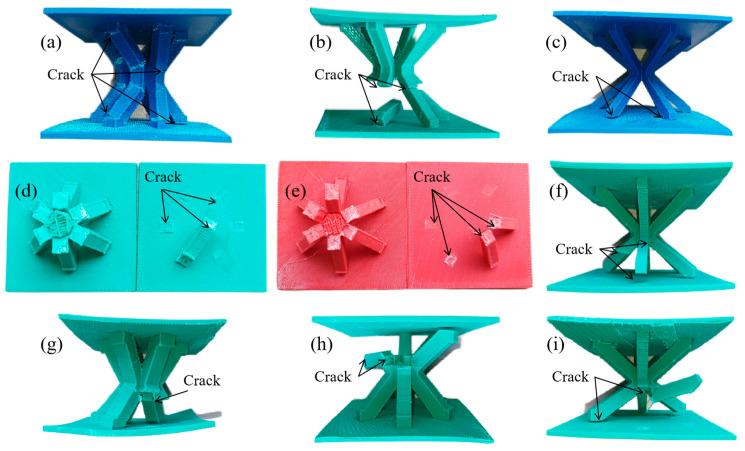
Compressed IFLS: (**a**) *θ* = 30°, *t* = 5 mm; (**b**) *θ* = 45°, *t* = 5 mm; (**c**) *θ* = 60°, *t* = 5 mm; (**d**) *θ* = 30°, *t* = 6 mm; (**e**) *θ* = 45°, *t* = 6 mm; (**f**) *θ* = 60°, *t* = 6 mm; (**g**) *θ* = 30°, *t* = 7 mm; (**h**) *θ* = 45°, *t* = 7 mm; (**i**) *θ* = 60°, *t* = 7 mm.

**Figure 18:**
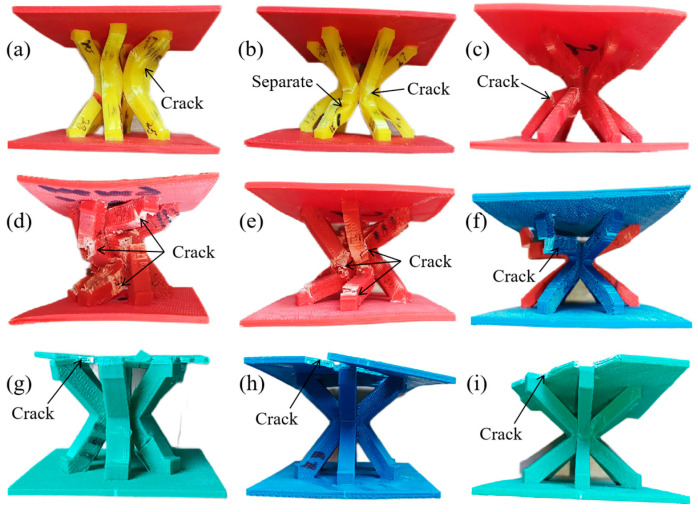
Compressed MTLS: (**a**) *θ* = 30°, *t* = 5 mm; (**b**) *θ* = 45°, *t* = 5 mm; (**c**) *θ* = 60°, *t* = 5 mm; (**d**) *θ* = 30°, *t* = 6 mm; (**e**) *θ* = 45°, *t* = 6 mm; (**f**) *θ* = 60°, *t* = 6 mm; (**g**) *θ* = 30°, *t* = 7 mm; (**h**) *θ* = 45°, *t* = 7 mm; (**i**) *θ* = 60°, *t* = 7 mm.

**Figure 19:**
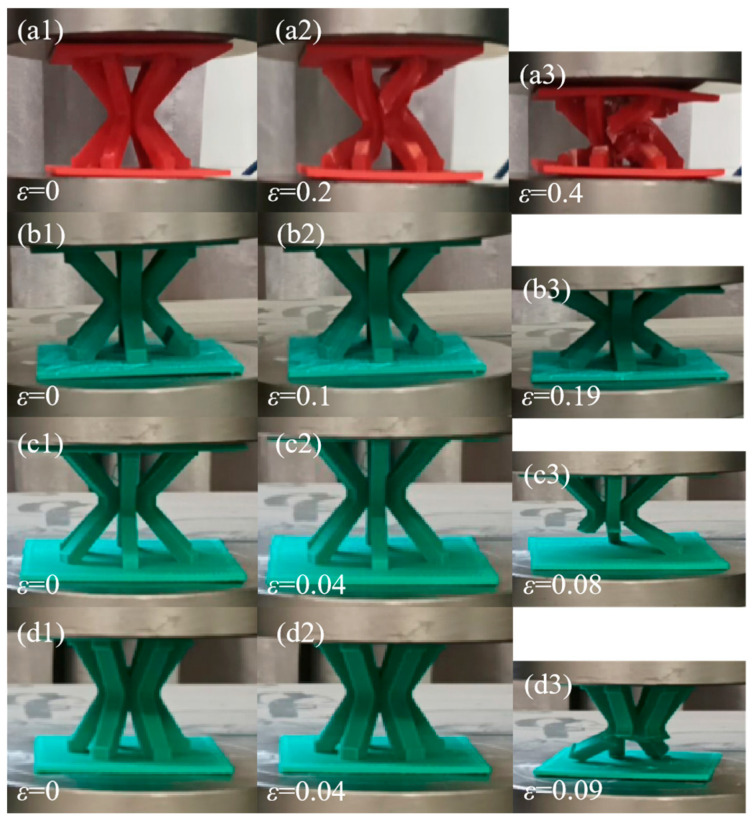
Images of lattice structure at different strains during compression: (**a1**) *ε* = 0 of *θ* = 45°, *t* = 6 mm MTLS; (**a2**) *ε* = 0.2 of *θ* = 45°, *t* = 6 mm MTLS; (**a3**) *ε* = 0.4 of *θ* = 45°, *t* = 6 mm MTLS; (**b1**) *ε* = 0 of *θ* = 45°, *t* = 7 mm MTLS; (**b2**) *ε* = 0.1 of *θ* = 45°, *t* = 7 mm MTLS; (**b3**) *ε* = 0.19 of *θ* = 45°, *t* = 7 mm MTLS; (**c1**) *ε* = 0 of *θ* = 45°, *t* = 5 mm IFLS; (**c2**) *ε* = 0.04 of *θ* = 45°, *t* = 5 mm IFLS; (**c3**) *ε* = 0.08 of *θ* = 45°, *t* = 5 mm IFLS; (**d1**) *ε* = 0 of *θ* = 30°, *t* = 6 mm IFLS; (**d2**) *ε* = 0.04 of *θ* = 30°, *t* = 6 mm IFLS; (**d3**) *ε* = 0.09 of *θ* = 30°, *t* = 6 mm IFLS.

The IFLS and MTLS after compression are shown in Figure 17 and Figure 18, respectively. Figure 19 shows the images of the lattice structure at different strains during the compression process.

**Figure 20:**
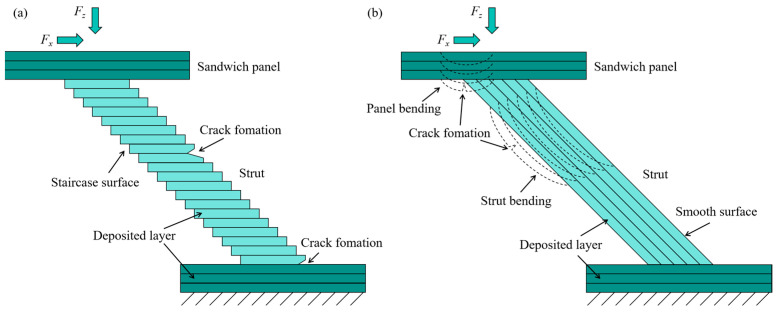
Schematic diagram of the failure mechanism of lattice structures: (**a**) IFLS; (**b**) MTLS.

As shown in Figure 17 and Figure 19(c1–c3,d1–d3), during the compression of the IFLS, the layered stacking between the struts and the sandwich plate causes a smaller contact area and weaker bonding strength, leading to crack initiation at the contact edges under compression. As strut deformation increases, debonding and fracturing occur between the struts and the sandwich plate. The struts experience shear stress under compression. Due to the weak bonding strength between the deposition layers, small deformations lead to cracks forming between two deposition layers. As the struts deform further, they fracture along the deposition layers, resulting in a very low stress plateau, as illustrated in Figure 20a.

As shown in Figure 18 and Figure 19(a1–a3,b1–b3), during the compression of the MTLS, increased strain causes the struts to rotate at the central nodes, forming plastic hinges. The struts bend under tensile stress at the plastic hinges, resulting in bending deformation. As the deformation of the struts increases, cracks form at the bending deformation regions, eventually leading to fracture. For MTLS struts with *θ* = 45° and *t* = 5 mm, bending deformation causes weak bonding strength between two deposition layers, resulting in delamination of the outer contour under shear stress. Because of the larger diameter of the struts, the MTLS with *t* = 7 mm experiences bending deformation of the sandwich plate as pressure increases. Cracks appear at the junctions between the sandwich plate and the struts under shear stress, eventually leading to fracture, while the struts themselves remain largely intact, as shown in Figure 20b.

### 3.4. Energy Absorption Properties

Lattice structures have excellent energy absorption characteristics. The total energy absorption (EA) refers to the total energy absorbed by the lattice throughout the compression process, whereas specific energy absorption (SEA) refers to the energy that the structure can absorb per unit mass. Based on the compression stress–strain curve, the specific expressions for EA and SEA in lattice structures are as follows:

(16) $$EA=\int_{0}^{\delta }Fd\delta$$

SEA = EA/*m* (17)

where *F* is the magnitude of the compressive force, *δ* is the final compressive displacement, and *m* is the mass of the structure.

**Figure 21:**
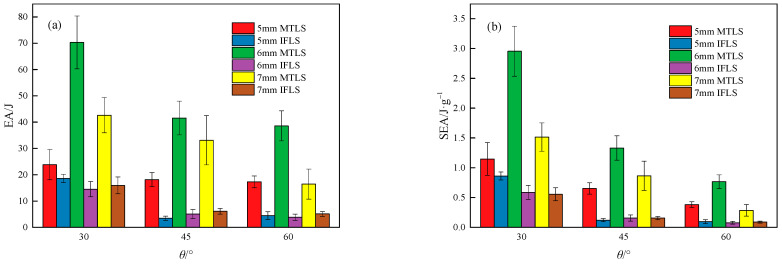
Comparison of energy absorption in lattice structures: (**a**) EA; (**b**) SEA.

The comparison of EA and SEA for different fabricated lattice structures is shown in Figure 21.

As shown in Figure 21, with the increase in *θ*, the EA and SEA of the lattice structures gradually decrease, except for the slight increase in EA for the 5 mm monolithic lattice structure at 45° and 60°. At *θ* = 60°, the EA and SEA of the MTLS decreased by 27.35%, 45.12%, and 61.45%, and by 66.71%, 74.06%, and 81.16%, compared with those at *θ* = 30°.

As *t* increases, the EA and SEA of the MTLS change significantly, first increasing and then decreasing; the EA and SEA of the IFLS change little. When *t* = 6 mm, the EA and SEA of the MTLS are the highest, with EA values of 70.31 J, 41.58 J, and 38.58 J and SEA values of 2.95 J/g, 1.33 J/g, and 0.77 J/g.

The EA and SEA of the MTLS are both greater than those of the IFLS, suggesting that the MTLS has superior energy absorption characteristics compared with the IFLS. Compared with the IFLS, the EA and SEA of the MTLS increased by 28.16% to 894.59% and 33.01% to 888.39%, respectively.

**Figure 22:**
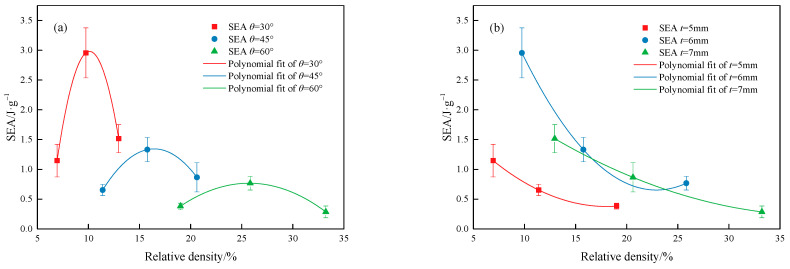
The relationship between the SEA of the MTLS and relative density: (**a**) SEA for different *t*; (**b**) SEA for different *θ*.

Figure 22 compares the relationship between the SEA of the MTLS and relative density. The relationship between SEA and relative density for the MTLS, as obtained from Figure 22, was fitted using a polynomial.

(18) $$\left\{\begin{array}{c} {SEA}_{30}=-0.18074{RD}^{2}+3.65608RD-15.51058\\ {SEA}_{45}=-0.02721{RD}^{2}+0.89394RD-6.00005\\ {SEA}_{60}=-0.00854{RD}^{2}+0.43943RD-4.88542 \end{array}\right.$$

(19) $$\left\{\begin{array}{c} {SEA}_{5}=-0.00622{RD}^{2}+0.22458RD+2.40363\\ {SEA}_{6}=-0.01331{RD}^{2}+0.60973RD+7.63373\\ {SEA}_{7}=-0.00191{RD}^{2}+0.14901RD+3.12436 \end{array}\right.$$

For the subsequent study, the equations can be used to derive the SEA of the MTLS at other relative densities. Therefore, these equations can aid in the design of lattice structures with better energy absorption in the future.

**Figure 23:**
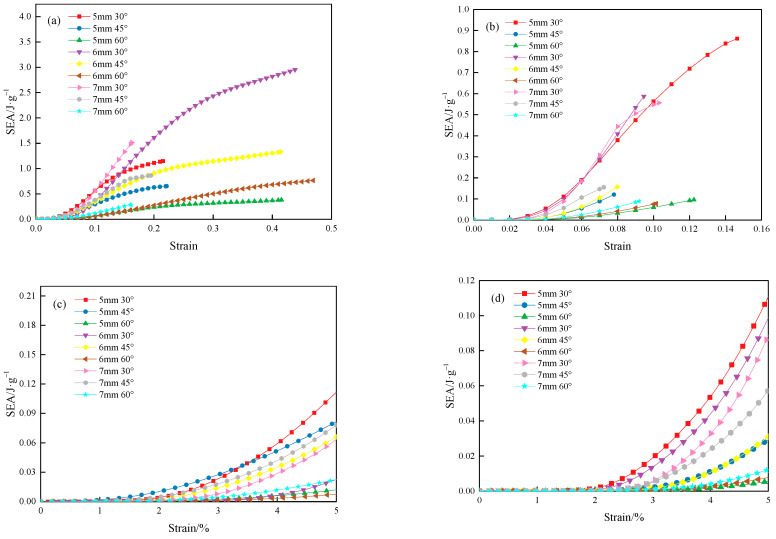
SEA–strain curve of the lattice structure: (**a**) MTLS; (**b**) IFLS; (**c**) *ε* = 0–5% MTLS; (**d**) *ε* = 0–5% IFLS.

Figure 23 shows the SEA–strain curve for the lattice structure. As seen in Figure 23, with the increase in strain, the SEA of the lattice structure gradually increases. In Figure 23a, after 10% strain, the SEA–strain curves for the MTLS can be classified into three distinct categories. The SEA–strain curve for *θ* = 30° is at the highest level, and the slope of the curve for *θ* = 30° and *t* = 7 mm is the steepest. For *θ* = 30° with *t* = 5 mm and 6 mm, and *θ* = 45°, the three curves are positioned in the middle. The three curves for *θ* = 60° are in the lower region, with the curve for *t* = 6 mm having the smallest slope.

Figure 23b shows that after 5% strain, the SEA–strain curves of the IFLS can be classified into three distinct categories. For *θ* = 30°, the three curves are at the highest range, with nearly identical slopes. The curves for *θ* = 45° occupy the middle range. The three curves for *θ* = 60° are at the lowest range, with the curve for *θ* = 60° and *t* = 5 mm having the smallest slope.

As seen in Figure 23c, during the initial compression stage (strain of 0–5%), the SEA–strain curves of the MTLS are categorized into two distinct types. The slope of the SEA–strain curve for the MTLS with *θ* = 30° and *t* = 5 mm gradually increases, reaching its peak at approximately 3.4% strain. For *θ* = 30° and *t* = 6 mm, as well as the three curves for *θ* = 60°, they are positioned at a lower range, with the curve for *θ* = 60° and *t* = 6 mm having the smallest slope. Notably, referring to Figure 23a, the energy absorption rate of the MTLS for *θ* = 30° and *t* = 6 mm increases as strain grows, exceeding the energy absorption of the lattice for *θ* = 30° and *t* = 5 mm at around 14% strain.

From Figure 23d, it can be seen that when the strain is between 0 and 5%, the SEA–strain curves of the IFLS are categorized into three distinct types, with the distribution pattern being consistent with that of the curves in Figure 23b. The curve with *θ* = 30° and *t* = 5 mm has the largest slope.

The SEA–strain curves of the MTLS and IFLS under the same *θ* demonstrate similar trends. As *t* increases, the slope of the curve increases slightly. This is likely due to the increased strut diameter, which makes the lattice structure less prone to bending deformation, resulting in stronger load-bearing capacity and improved energy absorption characteristics. As *θ* increases, the slope of the lattice SEA–strain curve gradually increases. This indicates that *t* has less impact on the energy absorption characteristics of the lattice structure; the lower the *θ*, the higher the SEA of the lattice structure. This is due to the increase in the cross-sectional area of the lattice as *θ* decreases, leading to a higher energy absorption capacity.

### 3.5. Discussions

The MTLS, inspired by traditional architectural joinery designs, is applicable to FDM technology. The fabricated MTLS struts are entirely formed in the X–Y plane without the use of support materials, exhibiting superior mechanical properties compared with traditional IFLS.

The anisotropy of the compressive performance of lattice structures is determined solely by the respective printing technology and is independent of the printing material. Therefore, it can be substituted with other thermoplastic materials, CF composites, or GF composites with higher strength and stiffness. Since AM is a layer-by-layer technology, other AM processes also exhibit mechanical anisotropy, which affects the compressive performance of lattice structures [57]. Consequently, the MTLS could be incorporated into other AM techniques, aiming to improve the compressive properties of lattice structures while lowering production costs.

When the thickness of the lattice structure struts is large, the thickness of the sandwich panel influences the mechanical properties of the lattice structure [58]. The failure mechanism shown in Figure 20b corresponds to an MTLS with a sandwich panel thickness of 2 mm. It can be observed that when the thickness is 7 mm, the sandwich panel undergoes bending deformation and fractures at the junction with the strut, while the strut itself remains largely intact. A thicker sandwich panel can better disperse and transfer external loads, reducing localized stress concentration in the lattice struts. When designing lattice structures, the sandwich panel thickness should be selected with consideration of the specific application scenario and energy absorption requirements to achieve an optimal balance between energy absorption and structural performance. In future work, compression tests on MTLS with thicker sandwich panels will be conducted.

## 4. Conclusions

To address the anisotropy of the FDM lattice structure and the challenges associated with removing support materials, this study introduces the traditional mortise and tenon joint process into FDM technology and designs a 3D MTLS based on a hexagonal dovetail. This method decomposes a 3D model into three 2D models, which are printed and then assembled. FDM technology was used to prepare MTLS and IFLS with different *θ* and *t* values. Compared with traditional integrated printing methods, the maximum reduction in side Ra for the MTLS under different *θ* values was 74.87%, with an average printing time saving of up to 25.44%, and an average material consumption saving of up to 50.16%. For different *t* values, the average printing time saving was up to 25.55%, and the average material consumption saving was up to 52.21%. Compared with IFLS, the compressive strength of the MTLS increased by 3.91–51.22%, and the compressive modulus decreased by 2.05–34.89%. EA increased by 28.16–894.59%, and SEA increased by 33.01–888.39%.

The effects of strut angle and strut thickness on the compressive performance of lattice structures were investigated. As *θ* increases, the strength, modulus, EA, and SEA of the MTLS gradually decrease. As *t* increases, the compressive strength and modulus of the MTLS gradually increase, while the specific strength and specific modulus remain almost unchanged. As *t* increases, the EA and SEA of the MTLS show significant variation, first increasing and then decreasing. The relationships between compressive strength, modulus, SEA, and relative density were predicted.

This study enhances the compressive properties of traditional IFLS and addresses the challenge of difficult support material removal, providing a novel solution for the fabrication of additive manufacturing lattice structures.

## Figures and Tables

**Table 1:** MTLS parameters.

No.	*t* (mm)	*θ* (°)	Relative Density (%)
1	5	30	6.93
2	5	45	11.39
3	5	60	19.01
4	6	30	9.75
5	6	45	15.76
6	6	60	25.83
7	7	30	12.96
8	7	45	20.62
9	7	60	33.23

**Table 2:** Performance parameters of ABS+ filament.

Density (g/cm^3^)	Diameter (mm)	Tensile Strength (MPa)	Elongation at Break (%)	Flexural Strength (MPa)
1.06	1.75	40	30	68

**Table 3:** Printing settings.

Nozzle Temperature (°C)	Bed Temperature (°C)	Layer Height (mm)	Print Speed	Infill Density (%)	Number of Outer Contours
270	90	0.25	Normal	100	2

## Data Availability

The original contributions presented in the study are included in the article; further inquiries can be directed to the corresponding authors.
